# Coupling Between Noise and Plasticity in *E. coli*

**DOI:** 10.1534/g3.113.008540

**Published:** 2013-10-11

**Authors:** Gajinder Pal Singh

**Affiliations:** School of Biotechnology, KIIT University, Bhubaneswar 751024, Odisha, India

**Keywords:** gene expression noise, expression plasticity, expression responsiveness, gene expression regulation

## Abstract

Expression levels of genes vary not only between different environmental conditions (“plasticity”) but also between genetically identical cells in constant environment (“noise”). Intriguingly, these two measures of gene expression variability correlate positively with each other in yeast. This coupling was found to be particularly strong for genes with specific promoter architecture (TATA box and high nucleosome occupancy) but weak for genes in which high noise may be detrimental (*e.g.*, essential genes), suggesting that noise–plasticity coupling is an evolvable trait in yeast and may constrain evolution of gene expression and promoter usage. Recently, similar genome-wide data on noise and plasticity have become available for *Escherichia coli*, providing the opportunity to study noise–plasticity correlation and its mechanism in a prokaryote, which follows a fundamentally different mode of transcription regulation than a eukaryote such as yeast. Using these data, I found significant positive correlation between noise and plasticity in *E. coli*. Furthermore, this coupling was highly influenced by the following: level of expression; essentiality and dosage sensitivity of genes; regulation by specific nucleoid-associated proteins, transcription factors, and sigma factors; and involvement in stress response. Many of these features are analogous to those found to influence noise–plasticity coupling in yeast. These results not only show the generality of noise–plasticity coupling across phylogenetically distant organisms but also suggest that its mechanism may be similar.

Expression levels of genes differ among genetically identical cells in the same environment. This variation or “noise” differs between genes, with some genes being noisier than others (Newman *et al.* 2006; Taniguchi *et al.* 2010; Silander *et al.* 2012). Cells also respond to different environmental conditions by changes in gene expression levels, with some genes showing higher expression responsiveness or “plasticity” than others (Tirosh *et al.* 2006; Choi and Kim 2009). These two measures of expression variation have been found to correlate in yeast (Landry *et al.* 2007; Lehner 2008, 2010; Choi and Kim 2009), suggesting that noise and plasticity are somehow mechanistically coupled (Tirosh *et al.* 2009). It was found that genes with TATA box promoters and high promoter nucleosome occupancy show much stronger coupling, suggesting the association with a particular mode of transcription regulation (Lehner 2010). Further, noise–plasticity coupling is lower for genes when it would be detrimental for fitness, *e.g.*, essential and dosage-sensitive genes (genes whose overexpression or underexpression inhibits growth) (Lehner 2010). These genes may need to have different responsiveness across conditions but cannot have very high noise, because this would lead to inappropriate expression levels in some cells.

It has been proposed that coupling between noise and plasticity should be a general feature of biological systems (Lehner and Kaneko 2011). In both bacteria and eukaryotes, mRNA production has been shown to occur in bursts, rather than in a continuous manner, which has been linked to expression noise (Golding *et al.* 2005; Kaern *et al.* 2005; So *et al.* 2011), suggesting that the source of transcriptional noise may be similar between bacteria and eukaryotes. Thus, noise–plasticity coupling observed in yeast may also be present in bacteria. Recently, promoter-mediated noise has been measured for a majority (60%, 1522 promoters) *of Escherichia coli* promoters (Silander *et al.* 2012) using a promoter library in which each strain carries a low-copy number (three to five copies per cell) plasmid with an *E. coli* promoter region inserted upstream of a gene for GFP. The expression variation of each gene among single cells was measured by flow cytometry. Given the strong correlation between mean expression level and expression variation, the expression variation for each gene was normalized to its mean, which was referred to as “noise.” These strains allowed the authors to ask whether promoter architecture influences noise–plasticity coupling in *E. coli*, independent of features like chromosomal context, mRNA half-life, translation, and protein half-life. Interestingly, noise–plasticity coupling was not observed in *E. coli* (Silander *et al.* 2012). The authors suggested that this may reflect a fundamentally different mode of transcription regulation between *E. coli* and yeast, *e.g.*, the lack of TATA box and nucleosomes in *E. coli*. Although there are no nucleosomes in *E. coli*, its genome is nevertheless highly compacted by nucleoid-associated proteins (NAPs) (Dillon and Dorman 2010), which may influence noise (Silander *et al.* 2012) and noise–plasticity coupling. I performed a more detailed analysis in *E. coli* and observed significant coupling. Importantly, noise–plasticity coupling was highly influenced by the mode and extent of transcription. The coupling was not observed in essential and slow-growth genes, but it was high in stress-responsive genes. These results are very similar to those in yeast, suggesting that noise–plasticity coupling may be common in organisms and its mechanism may also be similar.

## Materials and Methods

### Gene expression plasticity

Gene expression data were taken from the Many Microbe Microarrays Database (Faith *et al.* 2008), which contains uniformly normalized mRNA expression levels across conditions. All data in this database are from single-channel Affymetrix microarray only. For each gene, I measured the Standard Deviation (SD) of mRNA expression levels across different conditions (466 conditions; Build 6). The SD correlated significantly with mean expression (Pearson *r* = 0.40). Thus, to assess the expression variation independent of mean expression, I took residuals of the regression of the SD on mean as a measure of expression plasticity. This measure of expression plasticity did not show significant correlation with mean expression level (Spearman *P* = 0.3). As an alternate measure of expression plasticity, I calculated the distance of the SD of each gene from the running median with a window size of 200 (Supporting Information, Figure S1). The results remained essentially similar using this alternate measure of expression plasticity (Table S1).

### Gene expression noise

The promoter-mediated noise data for *E. coli* were taken from Silander *et al.* (2012). The authors of this study used an *E. coli* promoter library (Zaslaver *et al.* 2006), in which each strain carries a low-copy number plasmid (three to five copies per cell) with an *E. coli* promoter region inserted upstream of a gene for fast-folding GFP. Single-cell fluorescence measurements could be reliably performed for 1522 out of 1822 strains in the library. Most of the mRNA sequence is identical in each construct, allowing expression noise to be measured for different promoter sequences mostly independent of many other factors that are likely to affect protein expression noise, such as chromosomal context, mRNA half-life, translation, and protein half-life. There was a strong dependence of expression variation on mean expression level; thus, noise was defined as the deviation from smoothed spline of running median of expression level *vs.* coefficient of variation of expression. This metric decouples expression variation from mean expression level (Silander *et al.* 2012).

### Transcription-regulatory data

Transcription-regulatory data were taken from RegulonDB (Gama-Castro *et al.* 2011). All factors were analyzed in RegulonDB, which regulate 30 or more targets genes.

### Essentiality and dosage-sensitive and stress-responsive genes

Gene essentiality and growth data were taken from Baba *et al.* (2006). I defined slow-growth genes as those whose deletion leads to the slowest 10% growth rate (excluding essential genes). Overexpression toxicity data were taken from Kitagawa *et al.* (2005) and dosage-sensitive genes were defined by Singh and Dash (2013). Genes belonging to GO class “response to stress” were taken from ECOCYC (Keseler *et al.* 2013).

## Results

### Weak but significant correlation between noise and plasticity in *E. coli*

Noise–plasticity relationship has been investigated previously in *E. coli*, but no correlation had been observed (Silander *et al.* 2012). Authors of that previous study took expression data from an unpublished resource. These data consist of un-normalized expression data from different platforms and laboratories. Thus, it is possible that quality of expression data may be the reason behind lack of correlation observed by the authors. To test this hypothesis, I took a well-established resource in which expression levels are uniformly normalized and contain only single-channel Affymetrix microarray data (Faith *et al.* 2008). For each gene, I took the SD of mRNA expression levels across different conditions. The SD correlates significantly with mean expression levels (Pearson *r* = 0.40). To assess the expression variation independent of mean expression level, I took residuals of the regression of the SD on mean as a measure of expression plasticity. I found a weak but significant correlation between noise and plasticity (Spearman rho = 0.14; *P* = 8E−8; n =1456). As an alternate measure of expression plasticity, the distance between the SD of each gene from the running median of SD was calculated (see *Materials and Methods* section). This measure also indicated significant correlation between noise and plasticity (Spearman rho = 0.17; *P* = 1E−9). Thus, noise–plasticity coupling exists in *E. coli*, albeit weaker than in yeast (when Spearman correlation = 0.3 was observed) (Lehner 2010).

**Figure 1:**
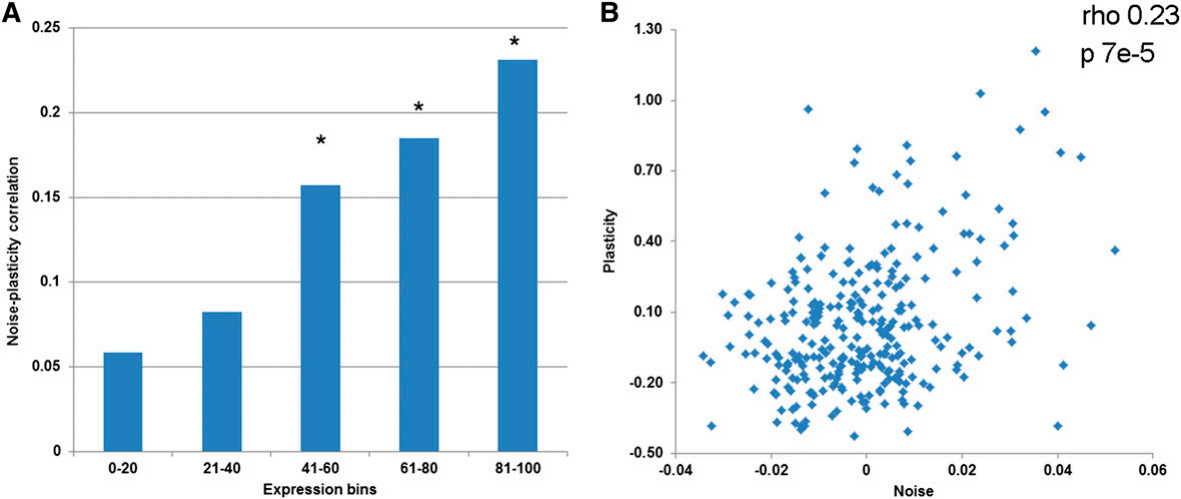
Noise–plasticity coupling for genes with different expression levels. (A) Spearman correlation coefficients between noise and plasticity are shown for genes divided into five equally populated nonoverlapping bins of genes according to their expression levels (*e.g.*, extreme left bin has 20% of lowest expressing genes). *Bins with significant correlation (*P* < 0.01). (B) Noise and plasticity values are plotted for the highest expression bin. Correlation and *P* are shown in the inset.

Next, I investigated whether level of expression might influence noise–plasticity coupling. Genes were placed into five equally sized nonoverlapping bins according to their expression level, and correlation between noise and plasticity was calculated for each bin. Genes with high mRNA expression levels showed high noise–plasticity coupling (genes with the top 20% expression show Spearman rho = 0.23; *P* = 7E−5; n = 291), whereas lowly expressed genes did not show significant coupling (Figure 1).

### Noise–plasticity coupling is disfavored for essential, slow-growth, and dosage-sensitive genes

In yeast, it was observed that noise–plasticity coupling was much lower for essential genes, genes whose deletion causes slow growth, and dosage-sensitive genes, but it was stronger for other genes (Lehner 2010). The noise in essential, slow-growth, and dosage-sensitive genes should be low and not coupled to plasticity, because this may lead to inappropriate (very low or very high) levels of proteins in some cells, which would be detrimental to fitness. Consistent with this, essential genes have low noise in yeast (Newman *et al.* 2006; Lehner 2008) and *E. coli* (Silander *et al.* 2012).

**Figure 2:**
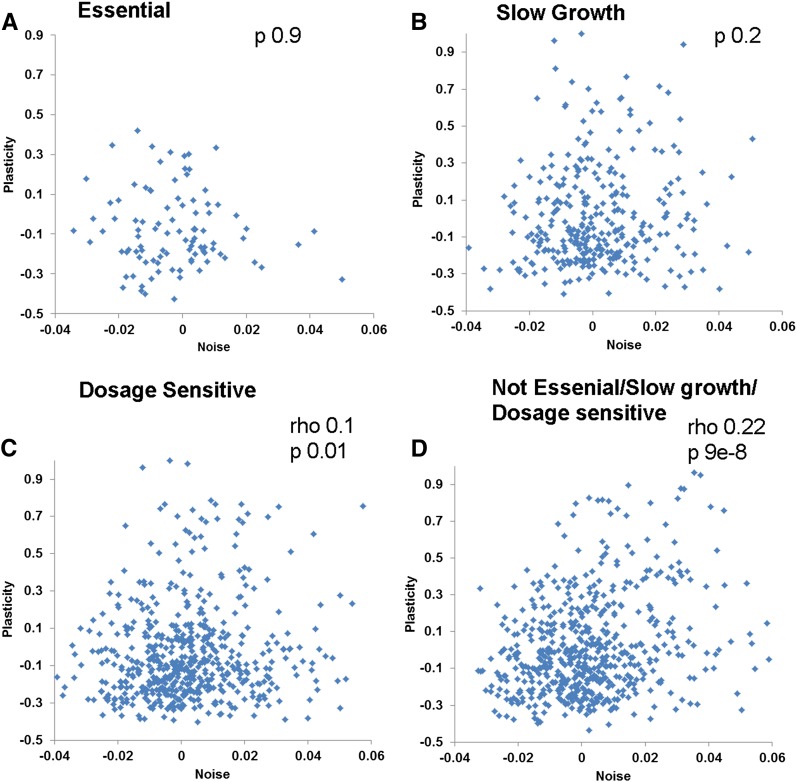
Noise–plasticity coupling for genes with different growth phenotypes. The correlation between gene expression noise and gene expression plasticity is shown for essential genes (A), genes whose deletion causes slow growth (B), genes whose overexpression is toxic (C), and nonessential, nonslow-growth, and nondosage-sensitive genes (D). Spearman rank correlation coefficients (rho) and *P* are shown in the inset.

I found no noise–plasticity coupling for essential (*P* = 0.87) and slow-growth genes (*P* = 0.19), and coupling was lower for dosage-sensitive genes (Spearman rho = 0.1; *P* = 0.01; n = 562), whereas in the other genes the coupling was stronger (Spearman rho = 0.22; *P* = 9E−8; n =574) (Figure 2). Even in highly expressed genes (which show stronger overall coupling), essential, slow-growth, and dosage-sensitive genes do not show significant coupling (*P* > 0.7), whereas highly expressed and nonessential, nonslow-growth, and nondosage-sensitive genes show further increases in coupling (Spearman rho = 0.46; *P* = 4E−7; n = 112).

Essential and slow-growth genes are expressed at significantly higher levels than the rest of the genes (two-tailed *t* test: *P* = 3E−18 and 0.006, respectively); therefore, the lack of correlation for these genes is not attributable to their different expression levels. Although dosage-sensitive genes are expressed at significantly lower levels, removing the lowest expressing dosage-sensitive genes (such that the mean expression of dosage-sensitive genes is not significantly different from the rest) did not change the noise–plasticity correlation (Table S2), indicating that the lower correlation of dosage-sensitive genes is not attributable to their lower expression level. In summary, these results are consistent with the model indicating that noise–plasticity coupling is an evolvable trait and natural selection can uncouple noise and plasticity when it is detrimental (Lehner 2010).

### Mode of transcriptional regulation influences noise–plasticity coupling

In yeast, it was observed that genes with a particular promoter architecture, specifically those with TATA box and high nucleosome occupancy, show much stronger noise–plasticity coupling (Lehner 2010). TATA box–containing promoters are frequently regulated by the SAGA complex and are associated with stress response (Blake *et al.* 2006). Although there are no equivalent TATA box promoters in *E. coli*, there are many global regulators (sigma factors, global transcription factors) whose transcription mechanism may be different from each other and may influence noise–plasticity coupling. Similarly, although there are no nucleosomes in *E. coli*, there are Nucleoid Associated Proteins (NAPs) such as H-NS (histone-like nucleoid structuring protein), Fis (factor for inversion stimulation), and IHF (integration host factor) that are involved in compaction of bacterial chromosomes and also influence gene expression (Dillon and Dorman 2010). Therefore, I tested whether different modes of transcription regulation influence noise–plasticity coupling.

**Figure 3:**
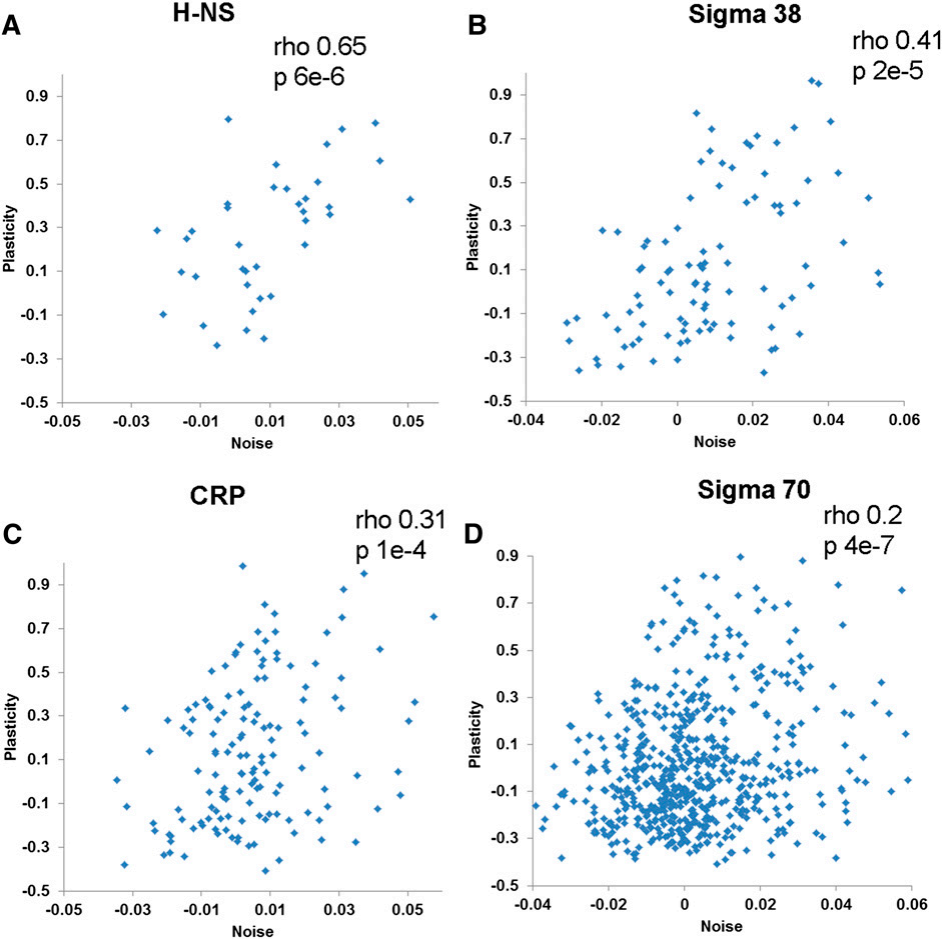
Noise–plasticity coupling for genes with different transcription regulation mechanisms. The correlation between gene expression noise and gene expression plasticity is shown for genes whose expression is regulated by H-NS (A), sigma 38 (B), CRP (C), and sigma 70 (D). Spearman rank correlation coefficients (rho) and *P* are shown in the inset.

Genes regulated by different regulators show marked difference in noise–plasticity coupling (Table 1), with H-NS–regulated genes showing the highest coupling (rho = 0.65; *P* = 4E−6; n = 42) (Figure 3A) and genes regulated by another NAP, such as IHF, showing no coupling (*P* = 0.43). Similarly, genes regulated by transcription factor CRP, sigma factor 38, and sigma factor 70 show high noise–plasticity coupling (Figure 3), whereas the rest of the global transcription and sigma factors do not show significant coupling (Table 1). Sigma 38 is the stress-response sigma factor in *E. coli*. To test whether high noise–plasticity coupling is a general feature of stress-responsive genes, I calculated the noise–plasticity correlation for genes in GO class “response to stress,” which are not known to be regulated by sigma 38. I found high coupling in these genes (Spearman rho = 0.34; *P* = 6E−5; n = 132). Conversely, sigma 38–regulated genes show high coupling even after excluding stress-response genes (Spearman rho = 0.40; *P* = 4E−4; n = 77).

**Table 1:** Coupling between expression noise and expression plasticity for different classes of genes in *E. coli*

Class	Spearman Correlation Coefficient (rho)	*P*	Genes
All	0.14	8.08E−08	1456
Highly expressed	0.23	7.27E−05	291
Essential	−0.01	0.94	99
Slow-growth	0.07	0.20	312
Dosage-sensitive	0.10	0.01	562
Not essential, slow-growth, or dosage-sensitive	0.22	8.53E−08	574
Highly expressed and not essential, slow-growth, or dosage-sensitive	0.46	4.20E−07	112
**NAPs**			
H-NS	0.65	6.56E−06	42
Fis	0.35	0.01	53
IHF	0.12	0.43	47
**Transcription factors**			
CRP	0.31	1.19E−04	147
ArcA	0.25	0.09	47
FNR	−0.01	0.93	74
FUR	0.31	0.09	31
**Sigma factors**			
Sigma 70	0.20	4.28E−07	634
Sigma 38	0.41	2.49E−05	100
Sigma 24	0.10	0.24	153
Sigma 28	0.30	0.03	51
Sigma 54	−0.01	0.97	30
Sigma 32	−0.11	0.25	120
**Stress response**	0.38	9.43E−07	155

There was no association between noise levels and noise–plasticity coupling for genes regulated by different regulators, *e.g.*, genes regulated by H-NS and IHF are significantly more noisy (Silander *et al.* 2012), but only H-NS shows high noise–plasticity coupling, suggesting that mode of transcription regulation, rather than noise levels of transcription itself, determines noise–plasticity coupling. Genes regulated by H-NS, sigma 70, and sigma 38 are not expressed at significantly higher levels than the rest of the genes (two-tailed *t* test: *P* > 0.12); therefore, high coupling for these genes is not attributable to their higher expression levels. Genes regulated by CRP, Fis, and stress-responsive genes are expressed at higher levels; therefore, I calculated noise–plasticity correlation after removing most highly expressed genes regulated by these regulators (thus eliminating the difference in expression level). For CRP, stress-responsive genes, and stress-responsive genes excluding sigma 38–regulated genes, the correlation remains high, but for Fis the correlation becomes nonsignificant (Table S2), suggesting that high coupling is independent of expression level for CRP-regulated genes and stress-responsive genes, but not for Fis-regulated genes. In summary, these results are highly analogous to those in yeast, for which the different mode of transcription regulation was associated with difference in noise–plasticity coupling.

## Discussion

Noise in gene expression levels and the responsiveness (plasticity) of gene expression to environmental changes are fundamental aspects of genes. Intriguingly, these two measures of gene expression variation were found to correlate in yeast (Landry *et al.* 2007; Lehner 2008, 2010; Choi and Kim 2009), although the mechanism of this coupling remains unclear. Recently, genome-wide promoter-mediated noise data for *E. coli* became available (Silander *et al.* 2012). However, the authors did not find significant correlation between noise and plasticity (Silander *et al.* 2012). I revisited this question using well-established and normalized gene expression data (Faith *et al.* 2008) to calculate expression plasticity and found that overall correlation between noise and plasticity is weak but highly significant (rho = 0.14; *P* = 8E−8).

I observed high noise–plasticity coupling for highly expressed genes, but not for lowly expressed genes (Figure 1). Noise in gene expression consists of two components, intrinsic noise and extrinsic noise (Elowitz *et al.* 2002). Intrinsic noise is attributable to the discrete nature of the biochemical process of gene expression and is the dominant source of noise for lowly expressed genes, whereas extrinsic noise is attributable to fluctuations in the levels of global factors that regulate gene expression, such as RNA polymerase, and is a dominant source of noise for highly expressed genes (Newman *et al.* 2006; Taniguchi *et al.* 2010). Different environmental conditions are likely to change the levels of global factors by changing growth rate and cell size (Klumpp and Hwa 2008); therefore, I speculate that high noise–plasticity coupling in highly expressed genes may be attributable to their high sensitivity to extrinsic noise. Interestingly, expression level was not found to influence noise–plasticity coupling in yeast (Lehner 2010), which may reflect difference in the regulation of global factors in *E. coli* and yeast.

Noise–plasticity coupling was not significant for essential and slow-growth genes and was weaker for dosage-sensitive genes (Table 1). Although the overall correlation was small, it nevertheless was enough for evolution to uncouple noise and plasticity for genes when it might be detrimental. Even when considering highly expressed genes (which show higher coupling), essential, slow-growth, and dosage-sensitive genes show no significant noise–plasticity correlation, but the rest of the highly expressed genes show a higher correlation of 0.46. These observations are consistent with the noise–plasticity coupling being an evolvable trait (Lehner 2010) in *E. coli*.

Although high coupling might be detrimental for dosage-sensitive and essential genes, it nevertheless might be advantageous for a subset of genes. This could be true for genes involved in the stress response, in which genes that are more responsive and allow physiological adaptation might also allow cell-to-cell variability, allowing some cells to survive harsh conditions. Genes regulated by stress-responsive sigma factor 38 and genes in GO class “stress response” show high noise–plasticity coupling (Table 1). This is analogous to TATA box genes in yeast, which are commonly associated with stress response (Blake *et al.* 2006) and show high noise–plasticity coupling (Lehner 2010).

Given the known influence of transcription mechanism on noise–plasticity coupling in yeast (Lehner 2010), I asked whether this is also true in *E. coli*. In *E. coli*, few global transcription regulators control the majority of regulated genes (Browning and Busby 2004). These include NAPs, which compact bacterial chromosomes and influence access to RNA polymerase and other transcription factors (Dillon and Dorman 2010). Genes regulated by different regulators show marked differences in noise–plasticity coupling (Table 1), *e.g.*, although H-NS shows high coupling, IHF does not. H-NS acts as a global transcriptional repressor and is important for forming topologically independent DNA microdomains (Hardy and Cozzarelli 2005). Similarly, genes regulated by transcription factor CRP show high coupling, whereas genes regulated by other global transcription factors do not show significant coupling (Table 1). It has been proposed that CRP may also act like NAP (Grainger *et al.* 2005). Consistent with this, genes repressed by CRP show much higher coupling (rho = 0.46; *P* = 0.002; n = 42) than genes activated by CRP (rho = 0.27; *P* = 0.003; n = 113). High nucleosome promoter occupancy has been associated with high noise–plasticity coupling in yeast. Our results show that, analogous to yeast, binding of specific NAPs to promoters is associated with high noise–plasticity coupling in *E. coli*.

Transcription regulation between prokaryotes and eukaryotes has been proposed to follow fundamentally different logic (Struhl 1999), and difference in noise–plasticity coupling between yeast and *E. coli* was proposed to reflect this difference (Silander *et al.* 2012). Our results regarding the influence of essentiality and promoter architecture on noise–plasticity coupling suggest that transcription regulation between eukaryotes and prokaryotes might be more similar than commonly appreciated. Indeed, mRNA production has been shown to occur in bursts in bacteria and in eukaryotes (Golding *et al.* 2005; Kaern *et al.* 2005; So *et al.* 2011). The burst-like transcription in TATA box–contacting promoters has been associated with high noise in gene expression in eukaryotes (Blake *et al.* 2006), which also show high noise–plasticity coupling. Thus, burst-like transcription might lead to noise–plasticity coupling in bacteria and eukaryotes. The fact that noise–plasticity coupling is present in yeast and in *E. coli* (and, hence, elephants) (Friedmann 2004) suggests that this may be a common feature of gene expression across organisms.

## Supplementary Material

Supporting Information
