# Supplementary material for: Coupling Between Noise and Plasticity in E. coli
Source: G3 (Bethesda). 2013 Oct 11;3(12):2115–20. doi: 10.1534/g3.113.008540 (PMC3852374; doi:10.1534/g3.113.008540)
Supplement: Supporting Information [file supp_3_12_2115__index.html]

Coupling Between Noise and Plasticity in E. coli — Supporting Information 

# Coupling Between Noise and Plasticity in *E. coli*

## Supporting Information for Singh, 2013

**Files in this Data Supplement:**

- Supporting Information - Figure S1 and Tables S1-S2 (PDF, 371 KB)
- Figure S1 - Correlation between mean expression level and standard deviation (SD) and calculation of expression plasticity metrics. (PDF, 215 KB)
- Table S1 - Coupling between expression noise and expression plasticity for different classes of genes in *E. coli*. (PDF, 300 KB)
- Table S2 - Coupling between expression noise and expression plasticity controlling for expression level. (PDF, 187 KB)
