# Supplementary material for: Coupling Between Noise and Plasticity in E. coli
Source: G3 (Bethesda). 2013 Oct 11;3(12):2115–20. doi: 10.1534/g3.113.008540 (PMC3852374; doi:10.1534/g3.113.008540)
Supplement: Supporting Information [file supp_g3.113.008540_008540SI.pdf]

G3· Genes | Genomes | Genetics

Coupling Between Noise and Plasticity in *Escherichia coli*

Gajinder Pal Singh
School of Biotechnology, KIIT University, Bhubaneswar 751024, Odisha, India
Tel : +91 674 2725732
Fax: +91-674-2725732
Email: gajinder.pal.singh@gmail.com

DOI: 10.1534/g3.113.008540

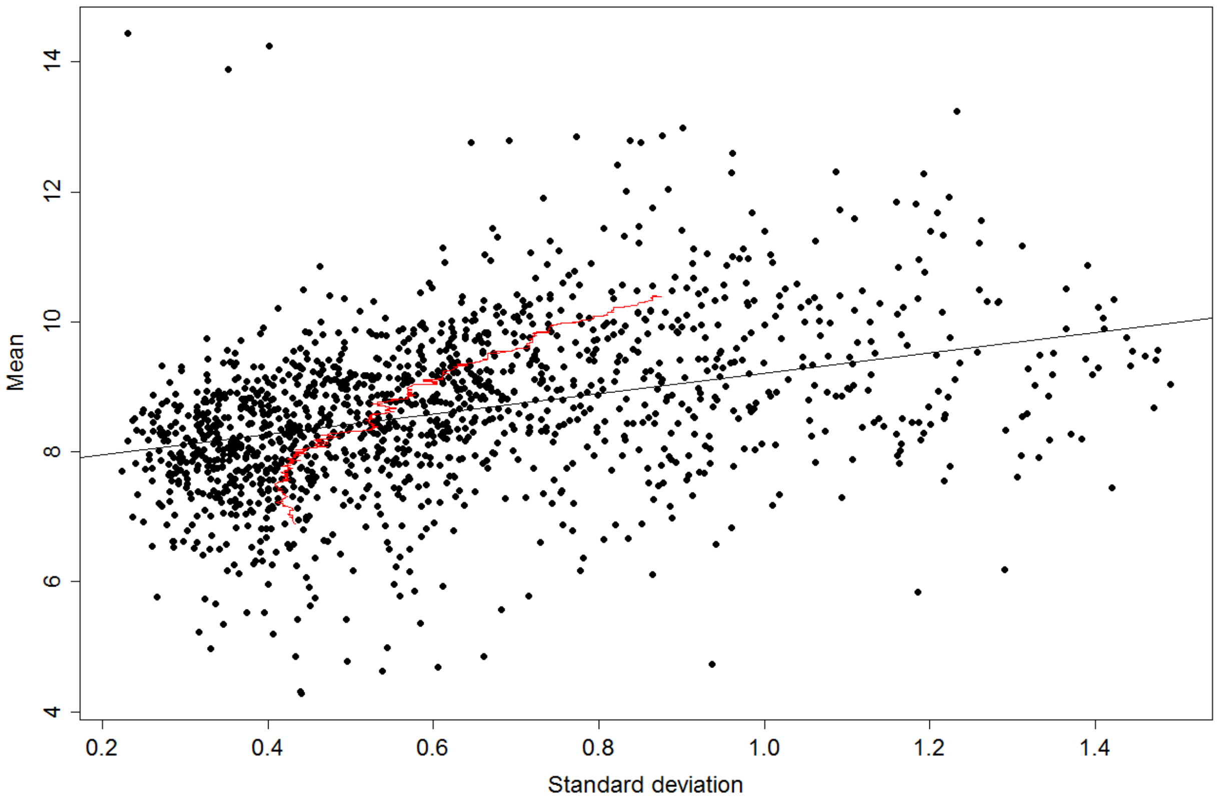

Figure S1 Correlation between mean expression level and standard deviation (SD) and calculation of expression plasticity metrics. The black line is the linear regression line. The residuals of SD from the linear regression line were taken as a measure of expression plasticity. The running median of SD is shown in red with a window size of 200 and truncated window-sizes at both ends. Distance of SD from running median was taken as an alternate measure of expression plasticity. The expression data was obtained from Many Microbe microarray database build 6 with gene expression values from 466 conditions.

Table S1 Coupling between expression noise and expression plasticity for different classes of genes in *E. coli.* Expression plasticity was measured by distance from the running median of standard deviation in the standard deviation versus mean expression plot (Figure S1).

Class	Spearman correlation coefficient (rho)	p-value	Genes
all	0.17	1.06E-09	1253
highly expressed (h)	0.31	4.68E-07	250
essential (e)	-0.02	0.89	69
slow growth (s)	0.11	0.07	257
dosage sensitive (d)	0.15	1.14E-03	494
not e,s,d	0.24	3.73E-08	504
h and not e,s,d	0.48	1.14E-07	108
**NAPs**			
H-NS	0.66	2.38E-05	34
Fis	0.24	0.13	40
IHF	0.08	0.64	38
**Transcription factors**			
CRP	0.29	7.65E-04	127
ArcA	0.23	0.17	37
FNR	0.06	0.62	62
FUR	0.36	0.08	25
**Sigma factors**			
Sigma70	0.21	1.00E-06	544
Sigma38	0.38	1.34E-04	95
Sigma24	0.11	0.20	133
Sigma28	0.33	0.02	48
Sigma54	0.09	0.66	27
Sigma32	-0.10	0.32	104
**Stress response**	0.39	5.64E-06	130

Table S2 Coupling between expression noise and expression plasticity controlling for expression level. Spearman correlation coefficients are shown for subset of genes in different classes which show no significant difference in expression level from rest of the genes. For dosage sensitive genes, 20% of genes with lowest expression are removed, while for genes regulated by CRP, Fis and stress responsive genes, 20% of highest expression genes are removed. Thus low noise-plasticity coupling of dosage sensitive genes, and high coupling for CRP and stress responsive genes (Table 1) is not due to difference in their expression level, while Fis no longer shows significant correlation, suggesting high coupling for Fis might be mediated by high expression of its target genes.

**Class**	**Spearman correlation coefficient (rho)**	**p-value**	**Genes**
Dosage sensitive	0.10	3.00E-02	450
CRP	0.29	1.45E-03	117
Fis	0.25	0.11	42
Stress response	0.41	2.46E-06	124
Stress response, not sigma38	0.37	1.23E-04	105
