# Supplementary material for: Coupling Between Noise and Plasticity in E. coli
Source: G3 (Bethesda). 2013 Oct 11;3(12):2115–20. doi: 10.1534/g3.113.008540 (PMC3852374; doi:10.1534/g3.113.008540)
Supplement: Supporting Information [file supp_g3.113.008540_FigureS1.pdf]

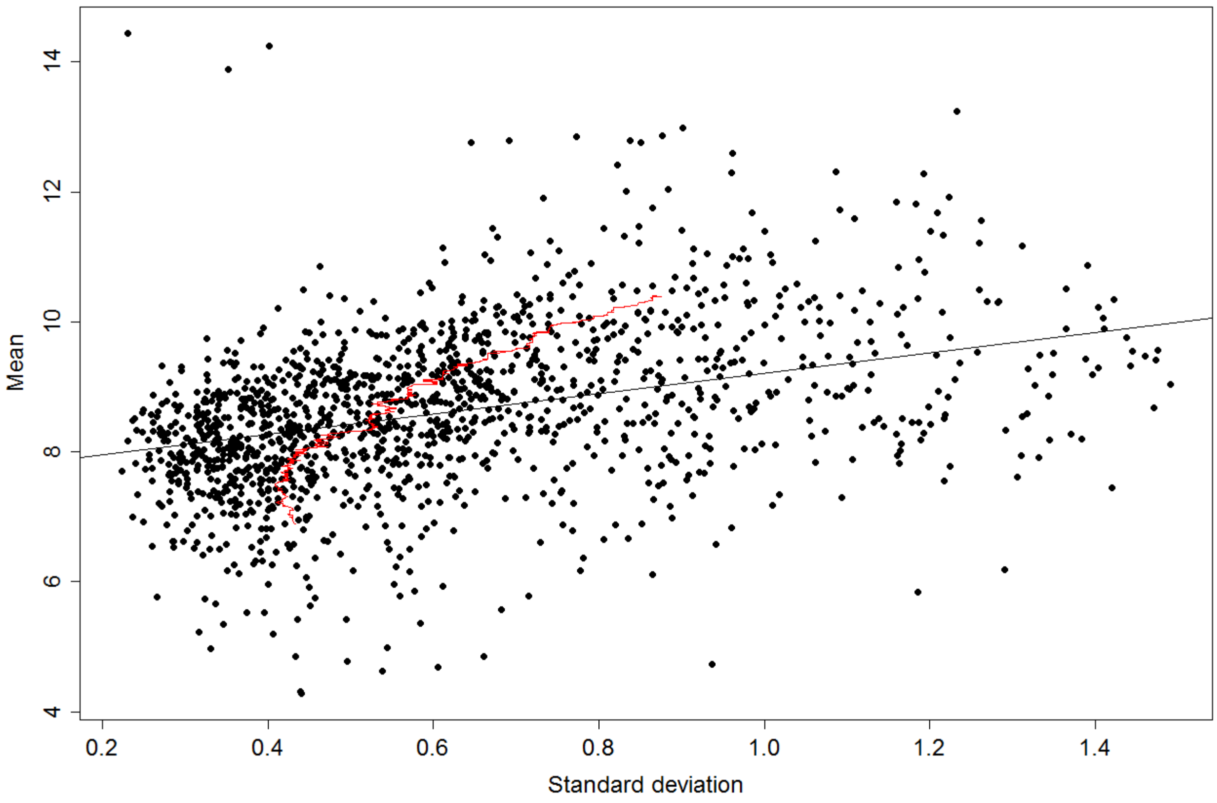

Figure S1 Correlation between mean expression level and standard deviation (SD) and calculation of expression plasticity metrics. The black line is the linear regression line. The residuals of SD from the linear regression line were taken as a measure of expression plasticity. The running median of SD is shown in red with a window size of 200 and truncated window-sizes at both ends. Distance of SD from running median was taken as an alternate measure of expression plasticity. The expression data was obtained from Many Microbe microarray database build 6 with gene expression values from 466 conditions.
