# Supplementary material for: Coupling Between Noise and Plasticity in E. coli
Source: G3 (Bethesda). 2013 Oct 11;3(12):2115–20. doi: 10.1534/g3.113.008540 (PMC3852374; doi:10.1534/g3.113.008540)
Supplement: Supporting Information [file supp_g3.113.008540_TableS1.pdf]

Table S1 Coupling between expression noise and expression plasticity for different classes of genes in *E. coli.* Expression plasticity was measured by distance from the running median of standard deviation in the standard deviation versus mean expression plot (Figure S1).

Class	Spearman correlation coefficient (rho)	p-value	Genes
all	0.17	1.06E-09	1253
highly expressed (h)	0.31	4.68E-07	250
essential (e)	-0.02	0.89	69
slow growth (s)	0.11	0.07	257
dosage sensitive (d)	0.15	1.14E-03	494
not e,s,d	0.24	3.73E-08	504
h and not e,s,d	0.48	1.14E-07	108
**NAPs**			
H-NS	0.66	2.38E-05	34
Fis	0.24	0.13	40
IHF	0.08	0.64	38
**Transcription factors**			
CRP	0.29	7.65E-04	127
ArcA	0.23	0.17	37
FNR	0.06	0.62	62
FUR	0.36	0.08	25
**Sigma factors**			
Sigma70	0.21	1.00E-06	544
Sigma38	0.38	1.34E-04	95
Sigma24	0.11	0.20	133
Sigma28	0.33	0.02	48
Sigma54	0.09	0.66	27
Sigma32	-0.10	0.32	104
**Stress response**	0.39	5.64E-06	130
