# Supplementary material for: Coupling Between Noise and Plasticity in E. coli
Source: G3 (Bethesda). 2013 Oct 11;3(12):2115–20. doi: 10.1534/g3.113.008540 (PMC3852374; doi:10.1534/g3.113.008540)
Supplement: Supporting Information [file supp_g3.113.008540_TableS2.pdf]

Table S2 Coupling between expression noise and expression plasticity controlling for expression level. Spearman correlation coefficients are shown for subset of genes in different classes which show no significant difference in expression level from rest of the genes. For dosage sensitive genes, 20% of genes with lowest expression are removed, while for genes regulated by CRP, Fis and stress responsive genes, 20% of highest expression genes are removed. Thus low noise-plasticity coupling of dosage sensitive genes, and high coupling for CRP and stress responsive genes (Table 1) is not due to difference in their expression level, while Fis no longer shows significant correlation, suggesting high coupling for Fis might be mediated by high expression of its target genes.

Class	Spearman correlation coefficient (rho)	p-value	Genes
Dosage sensitive	0.10	3.00E-02	450
CRP	0.29	1.45E-03	117
Fis	0.25	0.11	42
Stress response	0.41	2.46E-06	124
Stress response, not sigma38	0.37	1.23E-04	105
